# Variation in Body Mass and Skeletal Muscle Indices in Head and Neck Cancer Patients Undergoing (Chemo)Radiotherapy and Nutritional Intervention

**DOI:** 10.3390/curroncol30010020

**Published:** 2022-12-24

**Authors:** Carla Pisani, Federico Mastroleo, Alessandro Collo, Daniela Ferrante, Greta Carabelli, Pierfrancesco Franco, Sergio Riso, Valeria Dell’Era, Massimiliano Garzaro, Paolo Aluffi Valletti, Marco Krengli

**Affiliations:** 1 Division of Radiation Oncology, University Hospital “Maggiore della Carità”, Corso Mazzini 18, 28100 Novara, Italy; 2 Department of Translational Medicine, University of Piemonte Orientale (UPO), Via Solaroli 17, 28100 Novara, Italy; 3 Clinical Nutrition and Dietetic Unit, University Hospital “Maggiore della Carità”, Corso Mazzini 18, 28100 Novara, Italy; 4 Unit of Medical Statistics, Department of Translational Medicine, University of Piemonte Orientale (UPO) and Cancer Epidemiology, CPO Piemonte, Via Solaroli 17, 28100 Novara, Italy; 5 ENT Division, University Hospital “Maggiore della Carità”, Corso Mazzini 18, 28100 Novara, Italy

**Keywords:** head and neck cancer, radiotherapy, sarcopenia, nutritional assessment

## Abstract

The aim of this study was to analyze variation in body mass index (BMI) and skeletal muscle index (SMI) in head and neck squamous cell carcinoma (HNSCC) patients who underwent exclusive radiotherapy (RT) or concurrent chemo-radiotherapy (RT-CHT). We enrolled 73 HNSCC pts treated with definitive or post-operative RT (14 pts) or RT-CHT (59 pts). At the time of diagnosis (t_0_) and 3 months after treatment completion (t_3_), CT scans were retrieved to measure skeletal muscle at the level of the C3 vertebra. Median follow-up was 16 months. Nine disease progressions with distant metastases and eleven local relapses were observed. Fifty-three pts were free from progression at 1 year. At t_0_, average BMI was 25.8 (SD 4.1), while at t_3_ it was 24.5, with no reduction in 54 pts. A BMI decrease of −1.3 (*p*-value < 0.0001) between t_0_ and t_3_ was found with the Wilcoxon signed-rank test. SMI was 57.1 and 59.2 at t_0_ and t_3_, respectively (*p*-value = 0.005). According to our analysis, SMI variation seems to reflect the effect of an appropriate nutritional intervention and may represent a reliable, simple tool for muscle mass analysis.

## 1. Introduction

Head and neck squamous cell cancer (HNSCC) represents the sixth most common cancer in the world, accounting for nearly 65,000 new diagnoses and 350,000 deaths per year.

Due to an average age at diagnosis of 50 years old—with some differences related to primary tumor sites (oral cavity, pharynx, larynx, salivary glands) [1]—20% of HNSCC patients already suffer from several comorbidities which could impair treatment intensity, impacting clinical outcome [1,2].

Sarcopenia is a key factor that could further hamper treatment intensity in HNSCC patients [3], increasing chemotherapy-related toxicity and the rate of post-surgical complications, jeopardizing overall survival [4].

At baseline, the prevalence of sarcopenia in cancer patients ranges overall from 15% to 74% [4] and, according to a recent metanalysis, about 40% of HSNCC patients present with sarcopenia at diagnosis [5].

Aging-associated sarcopenia is known as primary sarcopenia, while secondary sarcopenia is related to sedentary lifestyle or inadequate nutrition [6]. Sarcopenia is a key component of cancer-related malnutrition, characterized by a loss of skeletal muscle mass and strength [7].

Skeletal muscle mass depletion in cancer is driven by enhanced intramuscular proteolytic system activity and triggered by a complex balance between reduced food intake, increased energy expenditure, systemic inflammation, tumor growth, and cancer therapy [8].

The 2019 Consensus of the European Working Group on Sarcopenia in Older People (EWGSOP) indicated reduced muscle strength, low muscle quality, and low physical performance as diagnostic criteria for sarcopenia [6].

This definition was based on studies performed on elderly patients and later translated to the cancer setting. Furthermore, recent cancer studies have started assessing sarcopenia by the measurement of muscle mass depletion using computed tomography (CT), introducing the adoption of prognostic cut-points to predict poor survival [7].

The assessment of the skeletal muscle index (SMI) at the level of the third lumbar vertebra (L3) is the gold standard for inferring total skeletal muscle mass through CT scans, and therefore to assess sarcopenia. In HNSCC patients, a positive correlation between skeletal muscle area (SMA) at the third cervical vertebra (C3: calculated using sternocleidomastoid and paravertebral muscles) and SMA at L3 has been demonstrated [9]. SMI is frequently used to assess and diagnose sarcopenia in clinical studies [10,11,12].

Nutritional interventions play a key role in maintaining and improving patients’ nutritional status, preventing skeletal muscle mass loss. According to current European Society for Clinical Nutrition and Metabolism (ESPEN) guidelines, all cancer patients should be assessed for malnutrition and followed up to perform the most appropriate nutritional strategy with oncological treatments, ranging from dietary counseling to medical nutrition. Early nutritional interventions include dietary advice, fortified foods, oral nutritional supplements (ONS), and artificial nutrition. [13].

Recently, new data have emerged in favor of the administration of oral glutamine (Gln) supplementation during RT or RT-CHT, since it could potentially reduce the severity of oral mucositis, preserve oral feeding (with natural foods plus/without ONS) and limit the need for artificial nutrition and/or treatment discontinuation.

Gln is the most abundant free amino acid with advantageous energetic, immune-modulatory, anti-inflammatory, and antioxidant properties. Low Gln levels were reported in HNSCC pts, and this deficient status is further worsened by anticancer therapy effects [14].

The aim of the present study was to retrospectively analyze a cohort of consecutive HNSCC patients who underwent RT or RT-CHT plus nutritional intervention, tracking their BMI and SMI variations between baseline and 3 months after treatment completion.

## 2. Materials and Methods

### 2.1. Study Population

We considered all HNSCC patients treated at our institution by RT or RT-CHT within a definitive or adjuvant setting between 2016 and 2020.

Inclusion criteria were HNSCC diagnosis, treatment with RT or RT-CHT, presence of a pre-treatment CT scan (CT t_0_) and a CT scan 3 months after treatment completion (CT t_3_) performed at our institution, presence of nutritional evaluation at baseline, as well as throughout and after the treatment. Patients without required clinical data, treated in a palliative setting, or with CT imaging performed outside our institution were excluded. For patients treated in an adjuvant setting, basal nutritional evaluation was performed before surgery. RT started within 6 weeks of surgery. Adopting these criteria, we finally included 73 consecutive patients in our analysis.

Clinical collected data were gender, age at diagnosis, tumor site, histology, grading and stage (AJCC 8th edition), HPV status, alcohol addiction (diagnosis of alcohol use disorder following DSM-V criteria), tobacco smoking habit, comorbidities (Charlson score), treatment details, treatment discontinuations, acute toxicities (CTCAE v 4.0 toxicity scale) at the beginning of treatment and after 3 months from its completion, and follow-up. Patients were followed-up by radiation oncologists and ENT surgeons.

Furthermore, nutritional collected data comprised weight, height, BMI, weight loss before diagnosis, presence and grade of dysphagia, and qualitative-quantitative assessment of dietary intakes (through 24 h recall technique performed by an expert dietician) at the beginning of the treatment and 3 months after treatment completion.

In order to meet metabolic needs, estimated through the ESPEN weight-based formula (25–30 kcal/kg/day for energy; 1.0 up to 1.5 g/kg/day for proteins) [3], an appropriate and personalized nutritional intervention was performed for all patients, ranging from dietary advice and fortified foods (oral intakes > 75% of energy requirements) to dietary advice plus oral nutritional supplements (ONS) (50–75%) to medical nutrition (<50%) [15].

Patients at risk of severe weight loss (5% weight loss over the last 1 month, or 10% over 6 months), inadequate caloric intake (<60% of TEE for more than 10 days), dehydration, severe dysphagia, or with predictable long-term swallowing disorders or severe pain which could have potentially impaired drink/food intakes, received pre-treatment prophylactic percutaneous enteral gastrostomy (PEG). All the other patients were treated by personalized dietary counselling ± ONS [16].

Gln was administered to all the patients enrolled after January 2018. Gln administration took place one week before RT start until one week after the end of the treatment. It was taken orally (or through PEG in cases of severe dysphagia) at a dosage of 21 g/day (one sachet 3 times a day).

Adjuvant RT started within 6 weeks of surgery. The time between surgery, post-operative re-habilitation, start of enteral nutrition, and RT was as short as possible to avoid a worsening of nutritional status and a worse oncological outcome, according to international guidelines.

The present study enrolled patients receiving standard-of-care treatments leading our local ethics committee to state that no specific formal ethics approval was required. In fact, our local ethics committee, “Comitato Etico Interaziendale Novara—AASSLL BI, NO, VCO, AOU “Maggiore della Carità” di Novara”, ruled that no formal ethics approval was required in this case because all the analyses were performed with no changes in patients’ treatments, but according to the best clinical practice. Our institution allows investigations into patients’ data upon the acceptance of radiotherapy informed consent. The study was performed in accordance with the Declaration of Helsinki.

### 2.2. SMI Calculation

**Figure 1:**
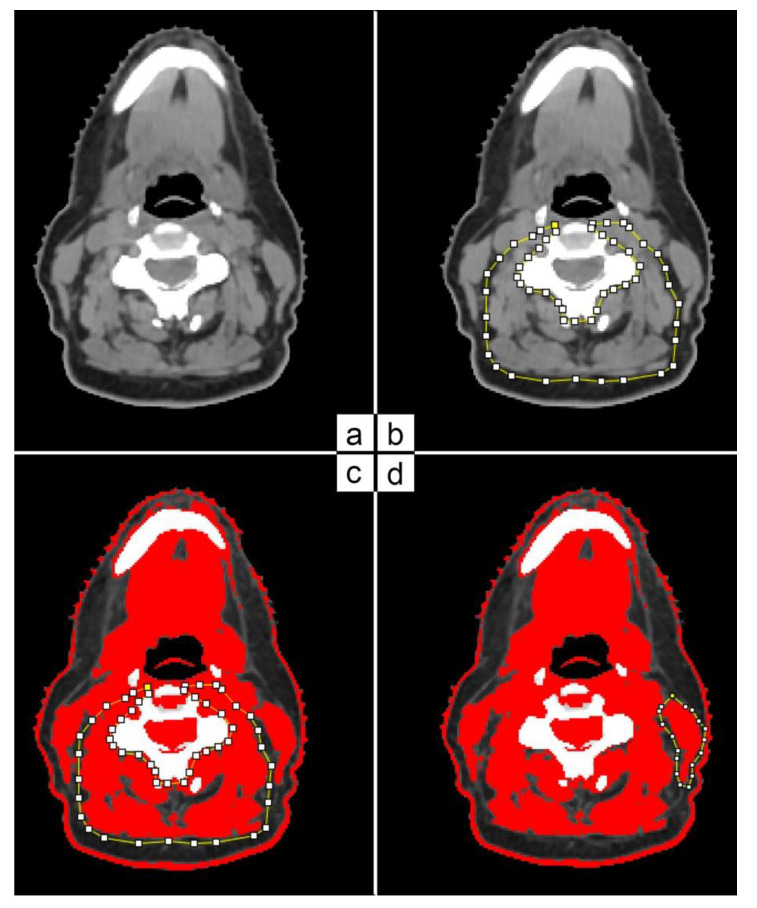
C3 paravertebral and SCM area delineation on CT scan based on HU threshold.

Cross-section area (CSA) was measured using a single slice at the level of C3 vertebra on patients’ t_0_ and t_3_ CT scans. The selected slice had to show the vertebral transverse process and the entire vertebral arc (Figure 1a). A resident radiation oncologist and an experienced radiation oncologist performed the muscle area delineation and analysis using ImageJ software (Figure 1b). Skeletal muscle area was defined as the pixel area between the radiodensity range of −29 and +150 Hounsfield units (HUs), encompassing muscles from very low and low density (−29 to +34 HU) to normal density (+35 to +150 HU) [17,18]. In the case of C3 paravertebral muscle areas (Figure 1c), the contralateral sternocleidomastoid (SCM) muscles were delineated (Figure 1d).

The CSA (cm^2^) of the skeletal muscle was then calculated as the sum of the areas of paravertebral muscles plus twice the value of a single SCM muscle.

*CSA at C3* (cm^2^) = *C3 paravertebral muscle area* + *one SCM muscle area* × *2*

We used a previously validated algorithm to estimate CSA at the L3 level.

*CSA at L3* (cm^2^) = *24.078 + 2.789 × CSA at C3* (cm^2^)

SMI was then calculated by further adjusting CSA at L3 for patients’ height (m^2^).

*SMI* (cm^2^/m^2^) = *CSA at L3* (cm^2^)/*Height2* (m^2^)

### 2.3. Statistical Analysis

Normally distributed data were presented as mean ± SD, whereas data following a non-normal distribution were presented as median + IQR. Categorical variables were summarized as counts and percentages. Differences in medians were evaluated using the Mann–Whitney U test and the Wilcoxon signed-rank test for pairwise comparisons. Associations between categorical variables were tested using the Pearson χ^2^ test or Fisher exact test, as appropriate. Disease progression or disease recurrence after complete remission were the events considered for progression-free survival (PFS). Analyses of patients’ survival followed Kaplan–Meier methodology with the log-rank test.

A two-sided *p* value < 0.05 was considered statistically significant. Analyses were performed using STATA software, version 17 (Stata-Corp. 2021. Statistical Software: Release 17.0. College Station, TX, USA: Stata Corporation) and MedCalc version 20.111.

## 3. Results

The mean age was 61 years with a prevalence of male gender (79.5%). At the time of diagnosis, 27/73 patients reported alcohol addiction (37.0%) and 61/73 tobacco smoking (83.6%). The Charlson score resulted in a median value of 4 [IQR 3–5]. Stage IV was the most common stage at diagnosis in 29/73 patients (39.7%). The histological type was squamous cell carcinoma in all cases. Oropharynx (37%) and oral cavity (21.9%) were the two most frequent sites of HNSCC. A descriptive analysis of patient and disease characteristics is reported in Table 1.

Eighteen patients (24.7%) were treated with the intensity-modulated technique (IMRT) and fifty-five (75.3%) with volumetric-modulated arc therapy (VMAT). In total, 59 patients (80.8%) received concurrent chemotherapy treatment with cisplatin: 31 (52.5%) with a weekly schedule (40 mg/m^2^) and 28 (47.5%) with a 3-weekly schedule (100 mg/m^2^). Twenty patients (27.4%) received post-operative radiation therapy. All patients received a baseline nutritional assessment. Fifty-two patients (71.2%) received early nutritional intervention and ten patients (13.7%) received PEG for nutritional therapy (integrative or exclusive enteral nutrition). Forty-two patients (57.5%), enrolled consecutively since January 2018, received oral or enteral Gln supplementation.

The most frequent grade for oral mucositis throughout the treatment and during the 3-month follow-up period was G2 (58.9%), followed by G3 (20.6%), G1 (12.3%), and G0 (8.2%). Similarly, the highest cutaneous acute toxicity was at G2 for 53.4% of patients, followed by G3 (21.9%), G1 (20.6%), and G0 (4.1%).

During treatment, 12 patients (16.4%) were admitted to hospital for G3 neutropenia, a sudden decrease in water or food intake, or impossibility of proceeding with an outpatient treatment. Seven out of twelve patients received Gln supplementation (58.3%), while three (4.1%) discontinued the treatment for toxicity prematurely, one of whom received Gln.

Mean BMI value was 25.8 ± 4.1 kg/m^2^ at t_0_ and 24.5 ± 3.6 kg/m^2^ at t_3_, with a significant decrease of 1.3 ± 1.8 kg/m^2^ (*p* < 0.0001).

Mean SMI value was 57.1 ± 11.0 cm^2^/m^2^ at t_0_ and 59.2 ± 11.8 cm^2^/m^2^ at t_3_, with an increase occurring in 46 patients (63.0%). SMI difference showed a significant increase of 2.0 ± 5.5 cm^2^/m^2^ at t_3_ (*p* = 0.005).

No significant results were found correlating BMI and SMI variation to other available variables (disease stage, Gln administration, smoking and alcohol addiction, acute mucosal, or skin toxicities). An extensive analysis report is available in Table 2.

**Figure 2:**
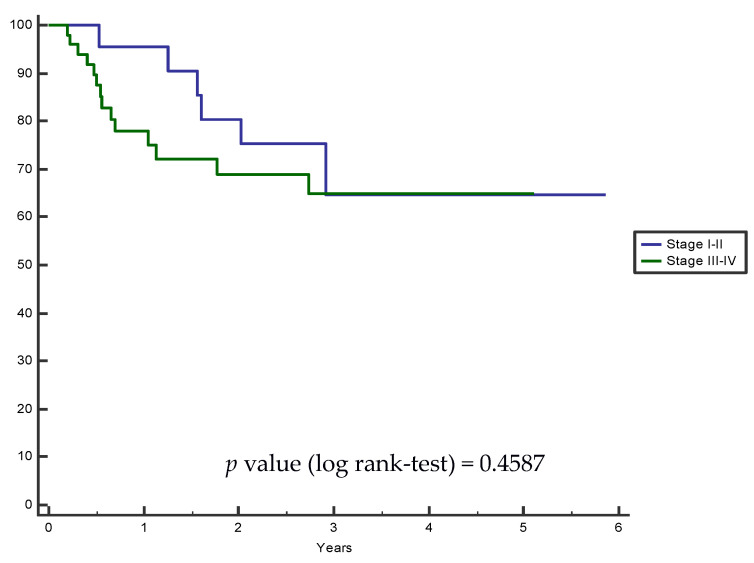
Kaplan–Meier progression-free grouped survival analysis: HNSCC stage I–II vs. III–IV.

Median follow-up was 22 months (range: 3–70 months). Kaplan–Meier progression-free survival analysis was performed in grouped stages (stage I–II vs. III–IV) and the log-rank test was not significant (Figure 2). Twenty patients experienced disease relapse (nine with disease progression with lung, hepatic or bone metastases, and eleven local relapses).

Three patients had treatment interruption, considered as the patient not receiving radiotherapy for more than 4 consecutive days. These three patients stopped treatment for a median of 7 days (SD ± 2.4 days). No significant difference was found between SMI variation and adverse events (*p*-value = 0.40).

Pearson’s chi-squared test did not show a significant association between Gln administration and mucosal toxicity (*p*-value = 0.15)

## 4. Discussion

Sarcopenia is a common wasting condition in cancer patients which leads to adverse prognosis and clinical outcome [1,3,4,5,17]. We did not stratify patients by TNM stages: all patients were evaluated at baseline, often before radiological staging definition. Early nutrition intervention was considered for all patients, regardless of staging, relying on weight loss and nutritional intake.

The early detection of sarcopenia is crucial to ensure timely and tailored nutritional support before an irreversible, refractory state takes hold. Although the role of nutritional intervention is well-established in HNSCC patients, its use in clinical practice is frequently neglected or delayed when oral mucositis or severe gastrointestinal reactions have arisen, potentially leading to restricted food intake. At this stage, nutritional treatment benefits are limited due to the intrinsic difficulty of improving patients’ nutritional status.

However, early nutritional intervention (dietary advice, fortified foods, oral nutritional supplements (ONS), and artificial nutrition) is not systematically included within conventional intervention strategies for HNSCC patients [19].

Thus far, BMI has had a fundamental role in nutritional assessment, being a commonly available tool. Previous studies failed to show a relationship between BMI and clinical outcomes in cancer patients, since weight and BMI fluctuations could hide an adiposity excess, body edema, or ascites [20,21]. Grossberg AJ et al. [22] reported that muscle mass loss represents a reliable prognostic factor for HNSCC patients, while weight loss alone is not significantly associated with overall survival. Further studies [23,24] confirmed that muscle mass depletion is associated with an increased risk of death and worse quality of life (QoL). This association is even stronger in cancer patients, who could experience higher RT-CHT toxicities and have a lower overall survival [10].

It is reasonable to consider that an adequate nutritional assessment should not be based only on absolute weight and BMI. In this sense, CT analysis of muscle mass, using SMI, is useful and feasible in an oncological setting, as CT scans are routinely performed during the diagnosis and staging of the disease, and body composition assessment requires approximately 20 min per scan with trained personnel [25].

Jung AR et al. [26] highlighted the tendency of a reduction in both SMI and BMI at 3 months from treatment completion in HNSCC patients, and a recent scoping review has confirmed this finding [27].

On the contrary, we observed a statistically significant SMI increment, probably due to an accurate nutritional assessment at baseline and during RT treatment. All patients also received a tailored nutritional intervention. Probably, all of these factors played a role in the increment of dietary intake and, consequently, raised SMI. Furthermore, we noticed that BMI variations during and after RT, in our study, were not related to nutritional intake.

Patients with stage I–II tumors showed a (not statistically significant) trend of higher BMI reduction compared with patients with stage III–IV. Notably, stage III–IV patients are more frequently malnourished and dysphagic at baseline, so more intensive treatments tend to be performed (from ONS to prophylactic PEG placement), resulting in improved nutritional status and body weight maintenance. At the same time, patients with stage III–IV tumors showed a smaller SMI increase, likely due to the advanced disease, worse general condition, performance status and higher refractoriness to the protein–anabolic stimulation of muscle mass. This reasonable hypothesis could potentially be confirmed by the assessment of inflammatory status (e.g., through PCR and interleukin determination). Current evidence shows that chronic low-grade inflammation, such as mucosal toxicity related to radiotherapy, actively contributes to the loss of muscle mass, strength and functionality, leading to sarcopenic status, especially in the elderly [28,29]. Inflammatory status assessment is missing in the present analysis, but it is our goal to evaluate it deeply in future studies.

Our analysis showed that patients who experienced G2/G3 acute skin or mucosal toxicity had a higher BMI decrease and SMI increase in comparison with patients with G0/G1, even though differences were not statistically significant.

Radiation-induced oral mucositis (RIOM) is a common RT side effect for HNC patients, reaching or exceeding a grade 3 toxicity in up to 66% of patients (Grade 3–4) [19,30].

However, in our population, acute mucosal toxicity was lower, as G3-grade RIOM was experienced in only 21.9% of patients, and no G4 events were observed. The most prevalent grade was G2 in 53.4% of cases.

This result could be due to different factors, such as the use of IMRT and VMAT techniques instead of 3D-conformal irradiation modalities. Moreover, appropriate and adequate nutritional support, early nursing, and medical care probably reduced toxicity.

The current literature underlines the importance of early nutritional intervention to improve HNSCC patients’ nutritional status, while limiting the incidence and severity of oral mucositis during RT or RT-CHT [18,31,32,33,34]. Thus far, the role of Gln supplementation remains an open issue and available evidence shows that it could perform anti-inflammatory and antioxidant action on muscle and mucosa, leading to therapeutic gain in structured nutritional interventions [14,33,35].

A direct association between SMI and Gln intake has not been demonstrated in our study, probably due to the retrospective design and the limited size and heterogeneity of the sample. Further prospective randomized studies of adequate sample size are needed to assess the role of Gln and confirm the recent literature findings.

We analyzed progression-free survival curves by grouped stages, and even if patients with stage III-IV had lower PFS rates than patients with stage I-II, the difference was not significant, but could be considered as slightly predictive with a *p*-value of 0.4587 (log-rank test). This finding is reported in some systematic reviews [5,11,12] which evaluated the relation between SMI, sarcopenia, and treatment outcome, confirming that sarcopenia is more frequently reported in locally advanced diseases, with an unfavorable prognosis. Furthermore, we are aware that patients with a higher disease stage could more often have a history of alcohol abuse and reduced nutritional intake, which could easily lead to a sarcopenic status.

We are aware that our study presents some limitations. Its major weakness is the heterogeneous enrollment (different tumor stages, tumor sites and treatment modalities) that could affect post-treatment nutrition status and muscle mass.

This is also a retrospective study, and the follow-up is relatively short. Taking into account the above-mentioned aspects, no statistical significance was reached. Moreover, some variables such as muscle strength and function, not necessarily correlated with muscle mass, have not been assessed, since to date there are no validated methods and cutoffs for cancer patients. Despite these limitations, patients enrolled in the study received similar treatment with IMRT and VMAT techniques, as well as a reproducible nutritional intervention based on current nutritional guidelines.

## 5. Conclusions

Our study shows that BMI variations do not correlate with muscle mass variations and nutritional intervention. On the contrary, we observed an increase in SMI values during and after RT treatment, probably due to the personalized nutritional assessment and intervention received by the undernourished HNSCC population.

In this uncertain setting, SMI can represent a reliable and feasible tool for muscle mass analysis that could easily be integrated in routine assessment, thanks to the use of already available resources, such as CT scans.

An early assessment of nutritional risk and a personalized nutritional intervention could allow HNSCC patients to achieve energy and protein targets during RT or CHT/RT, maintaining or improving muscle mass regardless of changes in BMI.

Further studies in wider and more select populations and a longer follow-up are needed to deeply investigate the possible correlations between SMI and clinical outcomes.

## Figures and Tables

**Table 1:** Study population characteristics: in the first column—clinical aspects related to gender, habits, histology, staging, and grading; in the second column—number of patients and percentage.

Feature/Variable	Number (%)
**Gender**	
Female	15 (20.5)
Male	58 (79.5)
**Age** (**mean** ± **SD**)	61 ± 10.9
**Alcohol use**	
No	46 (63.0)
Yes	27 (37.0)
**Smoking habit**	
No	12 (16.4)
Yes	61 (83.6)
**Charlson Score**	
1–4	45 (61.6)
5–11	28 (38.4)
**Tumor Site**	
Oral cavity	16 (21.9)
Oropharynx	27 (37.0)
Nasopharynx	14 (19.2)
Hypophpharynx	5 (6.9)
Larynx	9 (12.3)
Unknown primary	2 (2.7)
**HPV**	
Negative	27 (37.0)
Positive	26 (35.6)
NA	20 (27.4)
**Grading**	
G1	2 (2.7)
G2	32 (43.8)
G3	39 (53.4)
**Stage** (**AJCC 8th edition**)	
I	10 (13.7)
II	14 (19.2)
III	20 (27.4)
IV A	20 (27.4)
IV B	8 (11.0)
IV C	1 (1.4)

SD: standard deviation; HPV: positivity for papilloma virus infection; NA: not applicable/not reported.

**Table 2:** BMI and SMI variation in relation to patient and disease variables.

	BMI Difference T_3_ vs. T_0_ Mean ± SD (kg/m^2^)	SMI Difference T_3_ vs. T_0_ Mean ± SD (cm^2^/m^2^)
Gender		
Female	−1.5 ± 1.9	1.7 ± 5.0
Male	−1.3 ± 1.8	2.1 ± 5.6
*p*-value	0.96	0.86
Stage		
I–II	−1.5 ± 1.5	2.8 ± 4.7
III–IV	−1.2 ± 2.0	1.6 ± 5.8
*p*-value	0.51	0.25
Glutamine suppl.		
No	−1.4 ± 1.8	1.6 ± 6.0
Yes	−1.3 ± 1.9	2.4 ± 5.1
*p*-value	0.7378	0.4890
Smoking		
No	−1.8 ± 1.7	2.8 ± 4.9
Yes	−1.2 ± 1.8)	1.9 ± 5.6
*p*-value	0.2549	0.6233
Alcohol use		
No	−1.4 ± 1.7	1.7 ± 4.6
Yes	−1.2 ± 2.0	2.5 ± 6.8
*p*-value	0.5834	0.5002
Oral mucosal toxicity		
G0/G1	−0.8 ± 1.9	1.6 ± 5.1
G2/G3	−1.5 ± 1.8	2.1 ± 5.6
*p*-value	0.2486	0.8271
Acute skin toxicity		
G0/G1	−1.7 ± 1.1	1.2 ± 4.0
G2/G3	−1.2 ± 2.0	2.3 ± 5.9
*p*-value	0.1874	0.6450

## Data Availability

The data presented in this study are available on request from the corresponding author.
